# Percutaneous ultrasound-guided ulnar nerve release technique compared to open technique: A cadaveric study

**DOI:** 10.1051/sicotj/2022041

**Published:** 2022-09-26

**Authors:** Jad Mansour, Joe Ghanimeh, Abdelhamid Ghersi, Berenice Moutinot, Remy Coulomb, Pascal Kouyoumdjian, Olivier Mares

**Affiliations:** 1 Centre Hospitalier Universitaire Nîmes-Caremeau place du professeur Robert-Debré 30029 Nîmes France; 2 Department of Orthopedic Surgery, Lebanese American University-Rizk Hospital, Lebanese American University, School of Medicine Beirut Lebanon

**Keywords:** Ultrasound-guided ulnar nerve release – Cubital tunnel, Ulnar nerve release, Cadaveric

## Abstract

*Objectives*: To evaluate the outcomes of a novel percutaneous ultrasound-guided technique for release of ulnar nerve entrapment at the elbow when compared to standard open release *Methods*: One single surgeon performed an ultrasound-guided percutaneous release of the cubital tunnel on a group of five cadaveric elbows and open release on five others. All procedures were timed, and incision lengths were recorded. Meticulous anatomic dissection was then performed to assess the complete release of the carpal tunnel and iatrogenic injuries. *Results*: No significant difference was found between the two groups in terms of complete release and iatrogenic injury, whereas Operative time was significantly shorter for the US-guided technique. Incomplete releases of the nerve were found only during the first two trials in each group, while the third, fourth, and fifth trials showed a complete ulnar nerve release in both series, highlighting a fast learning curve for both techniques. All of this through a significantly smaller incision in the US-guided technique. *Conclusions*: This study highlights the similar effects of these two techniques in terms of complete release of the ulnar nerve, with no clear superiority of one over the other in terms of morbidity rate. Both have a fast learning curve for an ultrasound-trained surgeon, with the US-guided technique being a less traumatic and quicker alternative procedure.

## Introduction

Cubital tunnel syndrome (CuTS) is the second most common compression neuropathy affecting the upper extremity [1]. The symptoms of CuTS are usually paresthesia in the fourth and fifth fingers increasing during elbow flexion with associated elbow pain. In severe cases, motor deficits and intrinsic muscle atrophy of the hand can appear [2].

Treatment modalities for CuTS include non-operative interventions as the first line of treatment. If non-operative treatment fails, multiple surgical techniques exist, ranging from open surgical decompression [3] (with or without nerve transposition) [4] to endoscopic [5] to minimally invasive release [6].

Optimal surgical treatment of ulnar nerve entrapment at the elbow is still controversial as none of the techniques mentioned above has demonstrated clear long-term superiority over the others [7–9].

A novel percutaneous US-guided technique for the release of ulnar nerve entrapment at the elbow was described by Poujade et al. in 2014 [10]. This technique aims at releasing the different anatomic structures compressing the nerve while minimizing damage to the surrounding structures. Few subsequent papers revisited US-guided CuTS release, but no comparative study with the regular open approach is present in known literature [11–13].

Therefore, the primary aim of this study is to compare the ultrasound-guided release of the ulnar nerve (URUN) to the standard open release on 10 cadaveric elbows in terms of efficacy and complication rates. The secondary aims are to compare the operative time, learning curve, and incision length for these two techniques.

## Materials and methods

### Study design

This study was conducted at the Laboratory of Experimental Anatomy of the Faculty of Medicine of Montpellier-Nîmes, France. After approval by the ethical review committee of our institution, five fresh frozen cadavers were available, amounting 10 upper extremities. All specimens were adults (3 males and 2 females) between 42 and 71 (mean age = 59 ± 6) and free from any deformity or prior elbow surgery.

URUN was done on one upper extremity of each of the five cadavers, while the open release of the ulnar nerve (ORUN) was performed on the contralateral upper limb to reduce the risk of anatomical variation bias. All procedures were timed and performed by a single surgeon (JM). Following each procedure, incision length was recorded, and a second operator (co-author OM) performed meticulous anatomic dissection of the medial elbow to assess the release of the cubital tunnel and record possible iatrogenic injuries to the ulnar nerve itself or surrounding soft tissues, such as the flexor carpi ulnaris (FCU), ulnar artery, the medial antebrachial cutaneous nerve (MABC nerve) or basilic vein.

**Figure 1:**
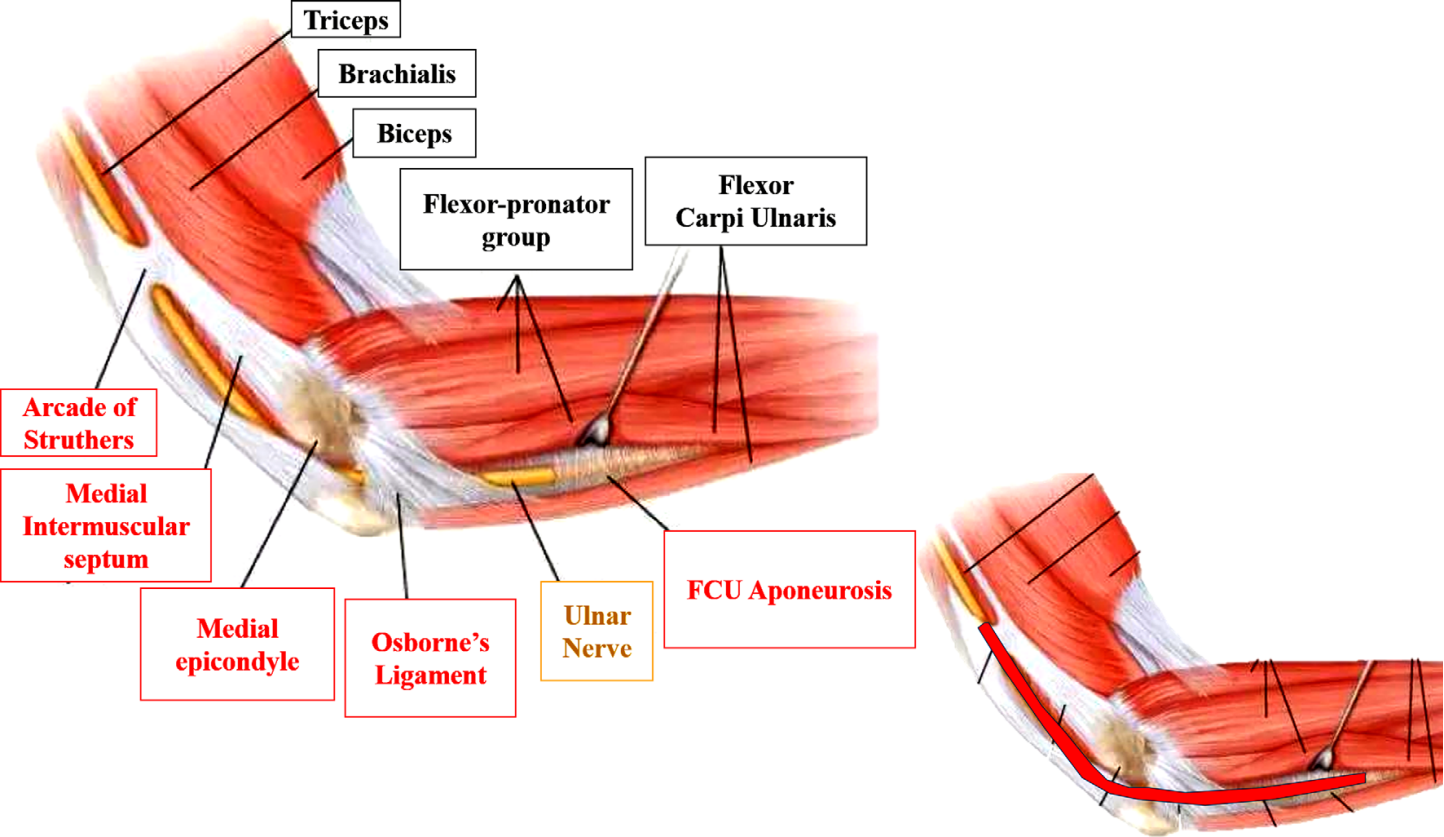
Schematic view of the medial elbow with possible sites of ulnar nerve compression (in red rectangles). At the bottom left, the release rate represents the percentage of the colored area’s length that was found to be sectioned upon dissection.

For the purpose of this study, the release rate was defined as the percentage of the longitudinal distance that was sectioned during the procedure between the arcade of Struthers proximally and the aponeurosis of the FCU distally. Figure 1 details the cubital tunnel, the most common possible sites of compression of the ulnar nerve, and the release rate.

The main outcome measures assessed were the release rate of the cubital tunnel and the iatrogenic injury rate. Secondary criteria selected were the operative time, learning curve, and incision length for each technique.

### Ultrasound-guided surgical technique

**Figure 2:**
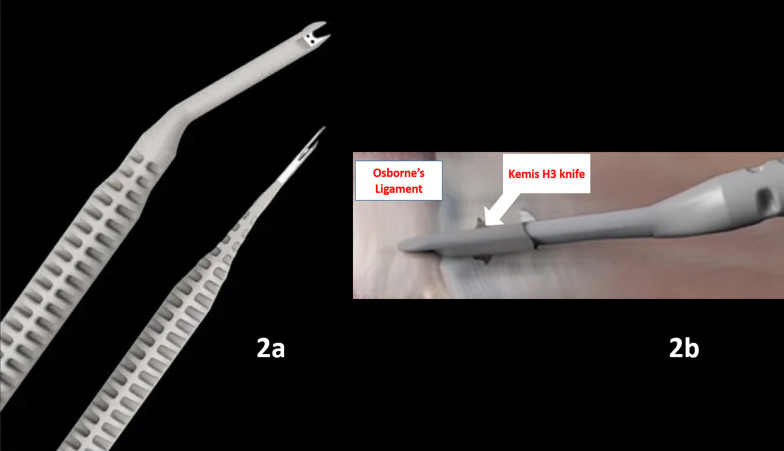
(a) Example of Kemis knives: on the left, the Kemis H3 blade used in the present study. On the right, the Kemis H1 blade can be used for treatment of trigger fingers or De Quervain tenosynovitis. (b) Illustration of the Kemis knife as used to transect Osborne’s ligament.

The ultrasound-guided procedures were performed using GE logiq ultrasound machine and 9/15 transducer type. The surgical dissection was performed through a millimetric skin incision using a KEMIS^®^ knife (Newclip^®^ Technics, France) (Figure 2). This type of knife has previously been used for the transection of the interosseous membrane of the forearm [14] and carpal tunnel release [15, 16] with success.

**Figure 3:**
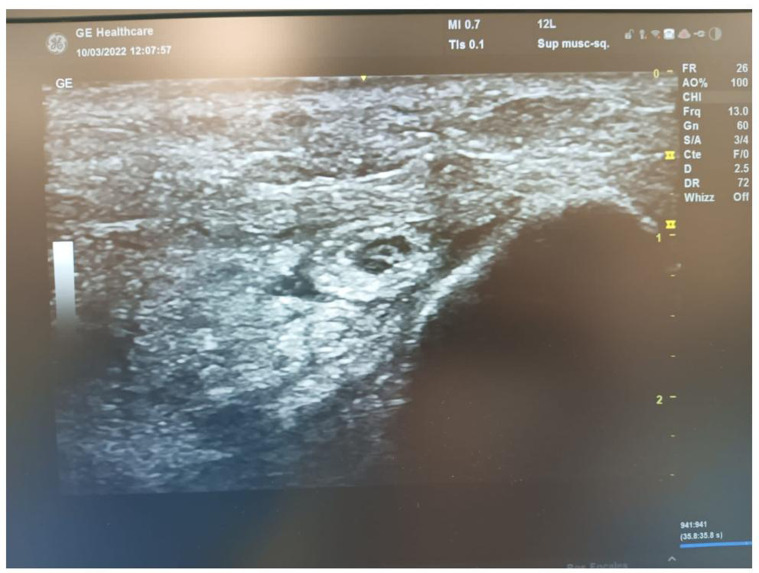
A transverse sonogram showing the compression site of ulnar nerve at medial elbow.

With the elbow on an arm board, we first start by identifying the ulnar nerve in its groove on both a longitudinal and transverse US view. Identification of the entry point at the distal aspect of the ulnar groove is then performed using an intramuscular needle. Following that, a few millimeters incision is done using a 15-blade, and subcutaneous tissue dissection is performed with curved Halsted forceps until the distal aspect of Osborne’s ligament is reached. The Kemis knife tip is then carefully inserted through our incision, and Osborne’s ligament is slid under US control between the two arms of the C-shaped blade (Figure 3). The latter is advanced through the cubital tunnel retinaculum towards the Struthers’ arcade, which is also released in the same fashion.

**Figure 4:**
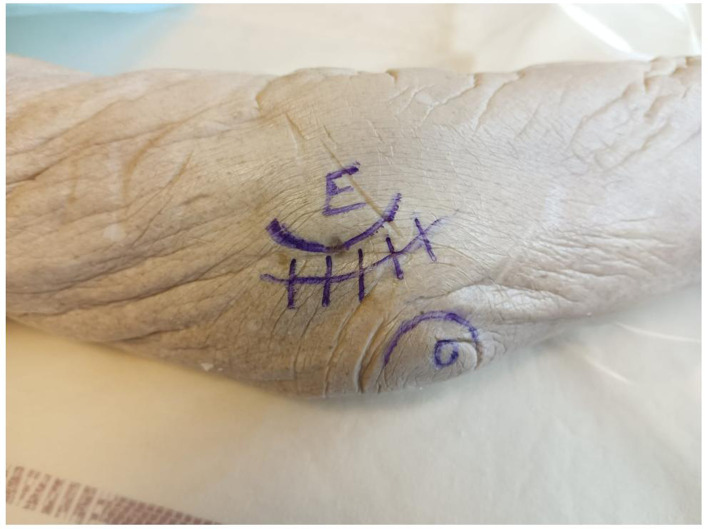
Incision of the open technique.

**Figure 5:**
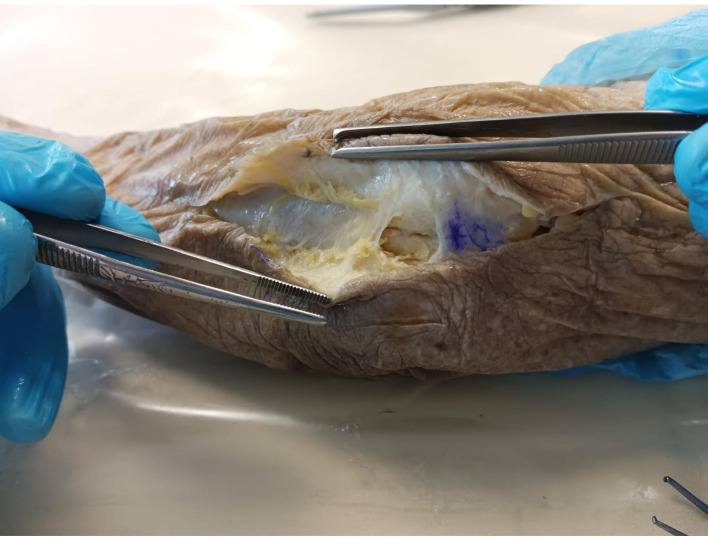
Sites of ulnar nerve compression.

Then, using the same entry point, the blade is reinserted and oriented distally in the same plane and advanced under US guidance towards the aponeurosis between the two heads of the FCU that can now be released as well.

A complete section of the cubital tunnel is finally assessed with the same curved Halsted forceps under US transverse views. The release was completed, if needed, with the knife again.

### Open surgical technique

The open technique consisted of dissection and identification of the ulnar nerve and *in situ* release of the cubital tunnel retinaculum, Struther’s arcade, Osborne’s ligament, and the aponeurosis between the two heads of FCU using a curved incision over the ulnar groove. (Figures 2 and 3).

### Statistical analysis

Qualitative variables were presented with their percentages. Quantitative variables were presented with their standard deviations and their minimum and maximum values. A Student’s test was used for the paired variables. The significance threshold was 5% for all the tests.

## Results

Section rates for both groups were comparable as the ultrasound-guided procedure had a mean rate of section of 0.97 ± 0.08 [0.88; 1], versus 0.93 ± 0.14 [0.93; 1] for the open release technique (*p* = 0.35). A complete section of the Osborne ligament and Struthers ligaments was consistently observed in both groups throughout the five trials.

The iatrogenic injury rate was also found to be comparable between the two groups since one case of FCU injury was noticed in each group, with no injuries in regards to other tendons, nerves (including the MABC and ulnar nerves), or vasculature in either group.

Operative time was shorter in the US-guided release group, with a mean duration of 16.6 min ± 6.9 [11; 24], versus 32 min ± 12.9 [29; 40] for the open release procedure (*p* = 0.014). The mean duration was divided by two between the first and the last cases in the ultrasound-guided technique, while it remained the same in the open release throughout.

Detailed data are reported in Tables 1 and 2 for the US-guided and open-release groups, respectively.

**Table 1:** Ultrasound-guided procedure outcomes.

Ultrasound-guided procedure (no)	1	2	3	4	5
Incision length (mm)	7	6	10	6	7
Cutting rate (%)	88	97	100	100	100
Duration (min)	24	22	14	11	12
Vascular injury	No	No	No	No	No
Flexor carpi ulnaris injury	Yes	No	No	No	No
Medial antebrachial cutaneous nerve injury	No	No	No	No	No
Osborne ligament release	Yes	Yes	Yes	Yes	Yes
Struthers ligament release	Yes	Yes	Yes	Yes	Yes
Flexor carpi ulnaris release	Yes	No	Yes	Yes	Yes

**Table 2:** Open release procedure outcomes.

Open release procedure (no)	1	2	3	4	5
Incision length	42	40	40	50	40
Cutting rate (%)	97	93	100	100	100
Duration (min)	29	34	32	40	25
Vascular injury	No	No	No	No	No
Flexor carpi ulnaris injury	No	No	Yes	No	No
Medial antebrachial cutaneous nerve injury	No	No	No	No	No
Osborne ligament release	Yes	Yes	Yes	Yes	Yes
Struthers ligament release	Yes	Yes	Yes	Yes	Yes
Flexor carpi ulnaris release	No	Yes	Yes	Yes	Yes

A fast learning curve was observed for both groups. Though incomplete section rates (and no release of the FCU aponeurosis) were noted in the first cases of both procedures, they systematically reached 100% of the cases as of the 3rd case, regardless of the procedure. The learning curve was thus set at four cases for the ultrasound-guided technique to obtain a rapid, efficient, and injury-free release.

Finally, the mean incision length of the ultrasound-guided procedure was smaller than for the open technique (7.2 mm ± 0.5 [6; 10] in the ultrasound group, versus 42.4 mm ± 8.1 [40, 50] in the open release technique one (*p* = 0.96).

## Discussion

Cubital tunnel syndrome affects 1.8–5.9% of the general population [17], with inconsistent success rates for conservative measures (splinting, activity modification, steroid injection) [18]. When the latter fails, and surgical treatment is warranted, a wide variety of surgical options are available, with no consensus as to which is the most efficient or reliable [8, 9].

In this series of 10 cadaveric elbows, we compared the outcomes of an URUN to those of the standard open release (ORUN).

The result of our study suggests that URUN at the elbow could be as effective and safe as the open technique, with a shorter operative time and a fast learning curve provided that the operator is familiar with ultrasound imaging, all this through a significantly smaller incision.

However, a major limitation of this study is that it is purely anatomical, and long-term cohort studies on symptomatic patients’ satisfaction are needed to confirm the superiority of URUN over ORUN. Moreover, the number of cadavers was limited to five for ethical considerations, and a larger number of procedures is needed to draw definitive conclusions as to the results mentioned above.

In this study, the total release rate was comparable for the two groups (95% and 100% in URUN and ORUN, respectively with no statistically significant difference (*p* > 0.05), while the number of iatrogenic lesions found after careful post-procedure dissection was identical in both series and similarly low. These findings highlight the similar effects of these two techniques in performing a complete release of the ulnar nerve, with none showing a clear superiority in terms of morbidity rate reduction.

These comparable outcomes were obtained with an operative time significantly shorter for the URUN group (16.6 min vs. 32 min), with the URUN being completed in nearly half as much time as the ORUN. This short operative time does not only favor URUN over ORUN but over endoscopic procedures as well, since the operative time of the latter varies in the literature between 35 min and 42 min [19, 20]. This advantage is significant when considering the cost of 1 min in the operating room is estimated to be 36–37$ [21].

Furthermore, incomplete releases of the nerve were found only during the first two trials of both groups, the fourth and fifth trials showing a complete ulnar nerve release in both series, suggesting a similarly fast learning curve for an ultrasound-trained surgeon. This conclusion was furthermore supported for the URUN group as operative time was divided by two between the first and last trial. Further studies are needed to validate the hypothesized fast learning curve of URUN in a large population of surgeons.

Finally, a statistically significant difference in incision length was noted, with the ORUN incisions being nearly six times longer than their URUN counterparts. This highlights the minimally invasive character of the URUN, implying less scarring of soft tissue and better aesthetics, and suggests the possibility of reduced pain and faster recovery in live subjects [22]. It is worth mentioning that incision length in URUN was even smaller than that of the endoscopic techniques (15–30 mm) [23–26], or the mini-open release (1.5–2.5 cm) [27], potentially making the URUN the least invasive procedure to date.

More than 40% of patients with ulnar nerve entrapment at the elbow will eventually need surgery [28]. The challenge when it comes to surgical treatment in CuTS, as in every surgical procedure, is to provide patients with the most efficient treatment while reducing unnecessary trauma to surrounding areas as well as operative time. From this perspective, many techniques have been described. First, the open release of the ulnar nerve allows direct visualization of compressive sites to ensure freedom of the nerve’s movement along its entire course [9]. Though it was found to be an effective technique with generally good results [29], complications such as superficial infections, wound dehiscence, incisional tenderness, and MABC nerve numbness was reported [30, 31], in addition to increased post-operative pain and delayed healing time [9]. The vast majority of surgeons still prefer open in situ decompression of the ulnar nerve at the elbow for its ease, low cost, and accessibility [32], with transposition progressively falling out of favor as multiple studies demonstrated an increased risk of complications with no added benefits [33–37].

The inconveniences of open ulnar nerve release led to the introduction of endoscopic decompression, first described in 1995 by Tsai et al. [5]. This technique appeared to offer the same results as open in situ decompression but with less scar tenderness [38], less intraoperative trauma [31, 39], fewer complications [40–44], faster recovery with an earlier return to work [26], at the expenses of a slightly longer operative time [8], an increased equipment cost and the need for more trained staff [9].

Mini-open cubital tunnel release was then introduced by Taniguchi et al. in 2022 in an attempt to get comparable results with the endoscopic technique through a 1.5–2.0 cm incision without the disadvantages of the latter [6]. The six subsequent studies conducted so far [20, 45–49] showed similar outcomes to the endoscopic technique in terms of patient satisfaction or complications, without the need for endoscopic equipment or an adequately trained team [27].

Later, in an effort to improve the effectiveness furthermore of in situ decompression while minimizing complication rates, URUN was developed by Poujade et al. in 2014 [10]. Ultrasounds are a cheap and radiation-free tool for anatomical and dynamic evaluation of upper extremity nerves and other soft tissues [50, 51]. Moreover, the anatomy of the ulnar nerve and its surroundings in CuTs is well documented and detailed in the scientific literature [52], with studies demonstrating its ability to identify and localize the compression better than electromyography [53, 54]. As such, this technology gained importance as a reliable complementary diagnostic tool for ulnar nerve entrapment [55–57]. The therapeutic role of US in CuTS has been traditionally limited to guiding corticosteroid injections [58, 59] in the same way it is used in other pathologies, such as lateral epicondylitis [60]. Some authors advocate for the use of US to better localize the site of compression pre-operatively and therefore target their decompression [61], however, the use of US as a guiding tool for real-time nerve decompression is still sporadic, and literature concerning URUN is still sparse.

Table 3 summarizes the current literature regarding CuTS release under ultrasound. To Note that all studies to date addressed the most common sites of ulnar nerve entrapment, but not post-traumatic etiologies or CuTS caused by abnormal muscle variants [62].

**Table 3:** Summary of current literature regarding US-guided CuTS release. NR: Not reported.

Study	Study design	Material used	Mean operative time	Mean Incision length	Outcomes	Limitations
Poujade et al. [10]	Cadaveric study on 16 elbows	Retractable Rosette blade	14.5 min	NR	No remaining compressive structures No iatrogenic injuries	– No comparison with a control group using another technique
				All were < 5 mm		
Boettcher et al. [12]	Case report on a 19-year-old professional swimmer	Stylet inserted in a spinal gauge and rotated to create a “V” cutting shape	NR	NR	Symptoms resolved Patient returned to sport at 2 weeks	Only the Cubital tunnel retinaculum was transected
Guo et al. [11]	Cadaveric study on 19 elbows	Percutaneous looped thread cubital tunnel release. Loop & Shear^TM^	20 min	No skin incision	Ulnar nerve was fully decompressed in all cases with no iatrogenic injury	– Only Osborne’s ligament and deep fascia were evaluated
						– No comparison with a control group using another technique
Kang et al. [13]	Cadaveric study on 29 elbows	Percutaneous looped thread cubital tunnel release. 3 types of dissecting threads were compared: Smartwire-01 Ultra V sswire Smartwire-01	NR	No skin incision	2/29 specimens were incompletely dissected No iatrogenic injuries	No analysis for common compression sites, such a Struther’s arcade or deep flexor pronator aponeurosis
Gruber et al. [64]	Case report on a 28-year-old road worker	Acufex hook knife	NR	NR	Symptoms resolved at 8 days post-op No iatrogenic injury	The Osborne’s ligament was the only structure to be transected

In the study by Poujade et al., percutaneous URUN was performed on 16 cadaveric specimens with no control group [10]. Similarly to our findings, no iatrogenic damage was noted on the nerves, and no remaining compressive structures were noted after URUN though fibers of one structure or more remained but with no evidence of true compression. Furthermore, URUN was found to carry the advantage of adapting each procedure according to the site of compression. Moreover, short operative times that were found to match those of the present study were noted with the same trend towards a shorter operative time between the first and last procedure, implying a fast learning curve for US-trained surgeons.

Ultrasound-guided ulnar nerve decompression may allow for a targeted and precise soft tissue release of the ulnar nerve while keeping important structures in sight. This application of ultrasonography could be an asset in the armamentarium of minimally invasive surgical treatment of CuTS. If further studies validate our findings, this procedure could be performed in ambulatory care or the interventional radiology room under local anesthesia and light sedation, similarly to what is done in ultrasound-guided carpal tunnel release [15, 16, 63].
